# The diagnostic value of glycated albumin in gestational diabetes mellitus

**DOI:** 10.1007/s40618-016-0605-7

**Published:** 2017-06-06

**Authors:** Jieping Zhu, Yu Chen, Changbin Li, Minfang Tao, Yincheng Teng

**Affiliations:** Department of Obstetrics and Gynecology, Shanghai 6th People’s Hospital, No 600 Yishan Road, Shanghai, 200233 China

**Keywords:** Fasting plasma glucose, Gestational diabetes mellitus, Glycated albumin, Glycated hemoglobin, Pregnancy

## Abstract

**Purpose:**

Our objective was to compare the diagnostic performance of glycated hemoglobin (HbA1c), GA, and fasting plasma glucose (FPG) for the diagnosis of GDM.

**Methods:**

Women at their late second or early third trimesters seen from October 2011 to April 2012 were studied. GDM was diagnosed based on oral glucose tolerance test results, and GA and HbA1c were measured at the same time. Patients were divided into two groups (with and without GDM), and areas under the receiver-operating characteristic curves (AUCs) were calculated to determine the diagnostic value of FPG, GA, and HbA1c.

**Results:**

A total of 698 women were included, of which 232 (33.2%) had GDM. Overall, FPG had the highest AUC for the detection of GDM, and was significantly higher than that of GA (0.692 vs. 0.568, *p* < 0.001) and HbA1c (0.692 vs. 0.619, *p* = 0.014). The AUC of FPG was significantly greater than that of GA and HbA1c. At 24–28 weeks’ gestation, the AUCs of FPG were significantly greater than those of GA and HbA1c.

**Conclusions:**

These results do not support the use of GA as a screening tool for GDM.

**Electronic supplementary material:**

The online version of this article (doi:10.1007/s40618-016-0605-7) contains supplementary material, which is available to authorized users.

## Introduction

Gestational diabetes mellitus (GDM), characterized by glucose intolerance that is first recognized during pregnancy, is associated with increased risks of macrosomia, shoulder dystocia and birth injuries, Cesarean delivery, premature delivery, and preeclampsia [1]. Furthermore, women with a history of GDM have been shown to have elevated catalase levels which positively correlate with glucose intolerance [2], and a tenfold increased risk of developing diabetes in the 10–20 years following pregnancy [3]. Children of mothers with GDM have an eightfold increased risk of developing type-2 DM during their lifetime [4]. The incidence of GDM has increased with the increasing rates of obesity and diabetes seen worldwide [5, 6], and new diagnostic criteria will result in GDM being diagnosed in approximately 18% of all pregnancies [7]. The diagnosis of GDM, however, is hampered by different criteria used internationally and across different institutions within the same country [8–10]. Furthermore, as pregnancy outcomes are worse for women with overt DM than for those with GDM, the World Health Organization (WHO) divides hyperglycemia in pregnancy as DM either preceding or first discovered during pregnancy, and GDM, i.e., hyperglycemia during pregnancy which resolves after pregnancy [11].

GDM is generally diagnosed with an oral glucose tolerance test, either as a 1- or 2-step procedure [12, 13]. However, testing is costly, requires multiple blood draws, and is susceptible to procedural variations, and borderline results require repeat testing. Glycated hemoglobin (hemoglobin A1c; HbA1c) is used to diagnose DM in non-pregnant individuals with a cut-off point of >6.5% considered diagnostic for DM [13]. While HbA1c can be used to estimate the risks of pregnancy complications [11, 14], it is not particularly useful for monitoring glycemic control during pregnancy as it reflects glucose control from 2 to 3 months prior, nor is it recommended for GDM screening [15]. Furthermore, HbA1c levels increase in the third trimester of pregnancy as a result of iron deficiency [16, 17].

Unlike HbA1c, glycated albumin (GA) reflects the mean blood glucose level in the prior 2–3 weeks, levels are not affected by albumin (Alb) concentration, and fasting is not necessary to perform the test [18]. While the use of HbA1c has extensively been studied in patients with DM [18–21], few studies have investigated its use in screening for GDM or monitoring patients with GDM [22–24]. A recent study specifically examining the value of GA in GDM reported that GA was less affected by insulin resistance and diastolic pressure than HbA1c, and the authors suggested that GA may be better than HbA1c for monitoring women with GDM [22]. A more comprehensive study from Japan Glycated Albumin (JGA) study group examined changes in GA and HbA1c in healthy pregnant women and reported that GA significantly decreased toward the third trimester, and was lower in women who were either obese or had proteinuria [24].

As GA has a large potential to be clinically useful in patients with GDM, the purpose of this study was to evaluate the diagnostic performance of GA in women with GDM, and compare the performance to that of HbA1c and fasting plasma glucose (FPG) level. We also sought to compare levels of these three markers in patients with and without GDM.

## Patients and methods

Pregnant women at their late second or early third trimester seen at the obstetrics department of our hospital during the period from October 2011 to April 2012 were prospectively recruitedfrom Shanghai 6th People’s Hospital. Patients with pregnancy complications other than GDM were excluded from the study. An age-matched control group of healthy women who were not pregnant was also included. This study was approved by the Institutional Review Board of Shanghai 6th People’s Hospital, and all patients provided written informed consent for participation in the study.

At the initial visit, FPG was measured to exclude pre-pregnancy DM. Other biochemical tests included Alb, alanine transaminase (ALT), aspartate aminotransferase (AST), creatinine (Cr), GA, hemoglobin (Hb), HbA1c, high-density lipoprotein cholesterol (HDL-C), low-density lipoprotein cholesterol (LDL-C), retinol conjugated protein 4 (RBP4), total bilirubin (TB), total bile acid (TBA), total cholesterol (TC), triglycerides (TG), total protein (TP), and uric acid (UA).

Glycated hemoglobin (HbA1c) was measured with an International Federation of Clinical Chemistry (IFCC) colorimetric method using a Sysmes XE-2100 device. The coefficient of variation of the assay was <3%. GA was measured with a chromatographic method using a Bio-radCobas-e 601 device (coefficient of variation <2%). All other tests were performed with a chemical spectrophotometric method using a Beckman AU5800 analyzer. Body weight was measured at different time points: at baseline and at 13–24, 24–28, and 32–36 weeks’ gestation. Body mass index (BMI) was calculated as body weight in kilogram divided by height in meters squared (kg/m^2^). GA and HbA1c were measured once on the same day as the OGTT. GDM was based on the definition of the American Diabetes Association (ADA) [12]. Briefly, pregnant women with an initial FPG ≥ 7.0 mmol/L, or HbA1c ≥ 6.5%, were diagnosed with the previous DM. FPG and HBA1c were measured in duplicate as suggested by the International Guidelines on diabetes diagnosis [7, 11, 13].

A 50 g glucose challenge test (GCT) was performed at 24–28 weeks’ gestation. If the plasma glucose 1 h after 50 g GCT was ≥7.8 mmol/L and ≤11.1 mmol/L, or if the plasma glucose one hour after 50 g GCT was ≥11.1 mmol/L, and FPG was <5.1 mmol/L, a 75 g OGTT will be ordered. If there is no indication of pre-existing DM, a 75 g OGTT is conducted at 24–28 weeks’ gestational age of to screen for GDM. If the 75 g OGTT is normal, but there is suspicion of GDM; it may be repeated in the third trimester. Plasma glucose levels measured fasting and 1 and 2 h after glucose intake ≥5.1, ≥10.0, and ≥8.5 mmol/L, respectively, are diagnostic of GDM.

Patients were divided into two groups based on the presence or absence of GDM diagnosed based on ADA criteria, and the diagnostic values of FPG, GA, and HbA1c for diagnosing GDM were examined.

## Statistical analysis

Normally distributed, continuous data were presented by mean ± standard deviation (SD), and differences between two groups were examined with the independent two samples *t* test. Non-normally distributed data were presented by median and inter-quartile range (IQR), and differences between two groups were examined with the Mann–Whitney test. Receiver-operating characteristic (ROC) curve analysis was performed to compare the diagnostic values of fasting plasma glucose (FPG), GA, and HbA1c. A higher area under the ROC curve (AUC) indicated a higher diagnostic value. Logistic regression analysis was performed to identify factors associated with GDM. Factors with significant associations in univariable logistic regression analyses were included in the multivariable logistic regression model according to the forward conditional method. All statistical analyses were performed with IBM SPSS statistical software version 22 (IBM Corp., Armonk, NY, USA). A two-sided *p* value <0.05 was considered statistically significant.

## Results

### Patient characteristics

From October 2011 to April 2012, 818 pregnant women were screened with an OGTT. Four had a history of DM, and 116 lacked data with respect to FPG, GA, HbA1c, or gestational weeks, and were, therefore, excluded from the analysis. Thus, 698 pregnant women were included in the study, and 232 (33.2%) were diagnosed with GDM. The control group consisted of 665 age-matched women who were not pregnant.

The characteristics of the patients with and without GDM are shown in Table 1. Women with GDM were older than those without GDM (*p* < 0.001), and had a higher BMI (*p* = 0.003). Women with GDM also had significantly higher TG (*p* < 0.001), RBP4 (*p* = 0.004), Alb (*p* = 0.007), FPG (*p* < 0.001), GA (*p* = 0.001), and HbA1c (*p* < 0.001) (Table 1).

**Table 1:** Characteristics of patients with and without GDM and control group

	Control (*n* = 665)	Case	
		Without GDM (*n* = 466)	With GDM (*n* = 232)
Age (years)	31.3 ± 5.7	29.3 ± 3.8^†^	30.9 ± 4.0*
BMI (kg/m^2^)	21.85 ± 2.8	20.9 ± 3.1^†^	21.6 ± 3.2*
ALT^a^ (U/L)	13.0 (11.0, 18.0)	16.0 (11.0, 25.0)^†^	15.0 (11.0, 26.0)^‡^
AST^a^ (U/L)	18.0 (16.0, 21.0)	19.0 (16.0, 24.0)	18.0 (15.0, 25.0)
TB (µmol/L)	13.0 ± 8.7	8.5 ± 2.8^†^	8.3 ± 3.0^‡^
TBA^a^ (µmol/L)	2.8 (1.7, 4.2)	2.0 (1.5, 3.0)^†^	2.0 (1.3, 2.8)^‡^
Urea^a^ (mmol/L)	–	2.6 (2.2, 3.0)	2.6 (2.2, 3.1)
Cr (µmol/L)	65.1 ± 11.5	43.0 ± 5.7^†^	42.9 ± 5.9^‡^
UA (µmol/L)	258.3 ± 55.9	205.0 ± 73.1^†^	212.0 ± 43.7^‡^
TC (mmol/L)	4.6 ± 2.8	5.1 ± 0.9^†^	5.2 ± 0.9^‡^
TG^a^ (mmol/L)	0.8 (0.7, 1.1)	1.4 (1.1, 1.8)^†^	1.6 (1.3, 1.9)*^,‡^
HDL-C (mmol/L)	1.5 ± 2.5	1.9 ± 0.4^†^	1.9 ± 0.4^‡^
LDL-C (mmol/L)	3.3 ± 10.4	2.6 ± 0.7	2.6 ± 0.7
Hb (g/L)	133.8 ± 39.8	115.5 ± 10.1^†^	116.1 ± 8.4^‡^
TP (g/L)	75.5 ± 4.4	67.5 ± 4.0^†^	68.1 ± 4.4^‡^
AG^a^	1.6 (1.5, 1.8)	1.7 (1.6, 1.9)^†^	1.8 (1.6, 1.9)^‡^
RBP4 (mg/L)	–	40.6 ± 6.8	42.3 ± 7.2*
Alb (g/L)	46.4 ± 3.3	42.6 ± 2.8^†^	43.2 ± 3.0*^,‡^
FPG (mmol/L)	4.9 ± 0.4	4.6 ± 0.3^†^	4.9 ± 0.5*
GA (%)	13.3 ± 1.2	11.8 ± 1.0^†^	12.1 ± 1.1*^,‡^
HbA1c (%)	5.3 ± 0.3	5.0 ± 0.3^†^	5.2 ± 0.4*^,‡^

Normally distributed data are presented by mean ± standard deviation

*AG* albumin/globulin ratio, *Alb* albumin, *Alt* alanine transaminase, *AST* aspartate aminotransferase, *BMI* body mass index, *Cr* creatinine, *FPG* fasting plasma glucose, *GA* glycated albumin, *GDM* gestational diabetes mellitus, *Hb* hemoglobin, *HbA1c* glycated hemoglobin, *HDL-C* high-density lipoprotein cholesterol, *LDL-C* low-density lipoprotein cholesterol, *RBP4* retinol conjugated protein 4, *TB* total bilirubin, *TBA* total bile acid, *TC* total cholesterol, *TG* triglycerides, *TP* total protein, *UA* uric acid

– indicates not available

*p < 0.05, Indicates statistically significant difference between with and without GDM

^†^ *p* < 0.05, Indicates statistically significant difference between control and without GDM

^‡^ *p* < 0.05, Indicates statistically significant difference between control and with GDM

^a^Non-normally distributed data are presented by median and inter-quartile range

Compared with the control group, the patients without GDM had significantly lower age, BMI (*p* < 0.001), TB (*p* = 0.008), TBA (*p* < 0.001), Cr (*p* < 0.001), UA (*p* < 0.001), Hb (*p* < 0.001), TP (*p* < 0.001), Alb (*p* < 0.001), FPG (*p* < 0.001), GA (*p* < 0.001), and HbA1c (*p* < 0.001); but had significantly higher TC (*p* < 0.001), TG (*p* < 0.001), HDL-C (*p* = 0.004), and AG (*p* < 0.001). The patients with GDM had significantly higher ALT (*p* < 0.001), TC (*p* = 0.002), TG (*p* < 0.001), HDL-C (*p* = 0.039), and AG (*p* < 0.001) compared with the control group, but significantly lower TB (*p* < 0.001), TBA (*p* < 0.001), Cr (*p* < 0.001), UA (*p* < 0.001), Hb (*p* < 0.001), TP (*p* < 0.001), Alb (*p* < 0.001), GA (*p* < 0.001), and HbA1c (*p* < 0.001) (Table 1).

Weight gain of women with and without GDM at different points in pregnancy is summarized in Supplemental Table 1. The mean weight and BMI were significantly higher in subjects with GDM compared with those without GDM before gestational week 38 to labor. There were no significant differences between subjects with or without GDM in weight gain from their pre-pregnancy weight.

### Diagnostic value of FPG, GA, and HbA1c

**Fig. 1:**
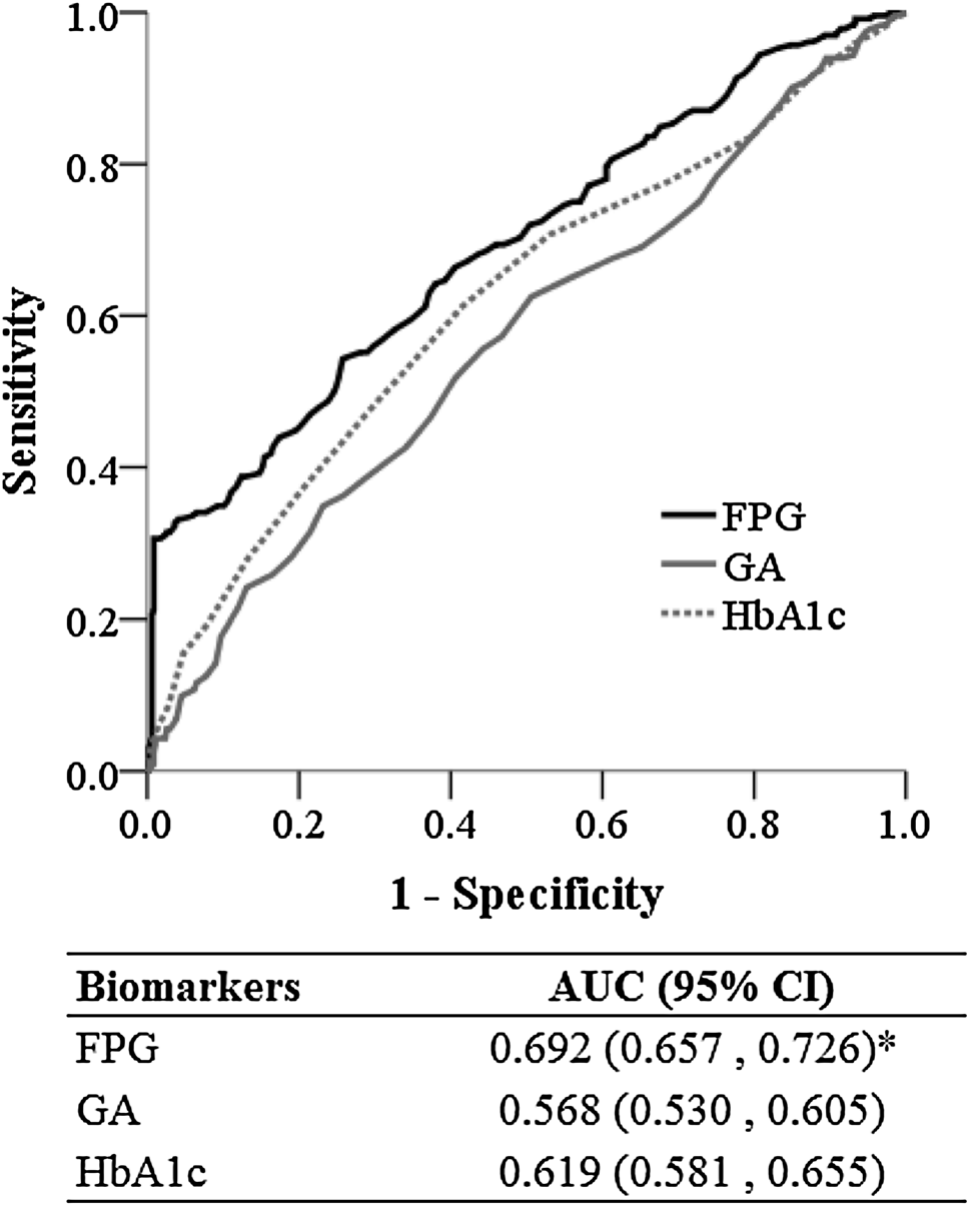
Diagnostic values of fasting plasma glucose (FPG), glycated albumin (GA), and glycated hemoglobin (HbA1c). *The area under the receiver-operating characteristic curve (AUC) of FPG was significantly greater than that of GA and HbA1c. *CI* confidence interval

FPG had the highest AUC for the detection of GDM, and was significantly higher than the AUCs of GA (0.692 vs. 0.568, *p* < 0.001) and HbA1c (0.692 vs. 0.619, *p* = 0.014) (Fig. 1). There was no difference in the AUCs of GA and HbA1c. These results indicate that the diagnostic value of FPG is greater than that of GA and HbA1c.

### Factors influencing GDM

Univariable logistic regression analyses showed that women were more likely to have GDM if they were older, had a greater BMI, and had higher TG, RBP4, ALB, FPG, GA, and HbA1c levels (Table 2). The associations of BMI, TG, and RBP4, however, did not remain statistically significant in multivariable analysis and were thus excluded from the final multivariable model. The final multivariable model showed that older age and higher levels of Alb, FPG, GA, and HbA1c were significantly associated with GDM. The odds of having GDM were increased with every 1 year increase in age [odds ratio (OR) = 1.09, *p* < 0.001], with every 1 unit increase of Alb (OR = 1.08, *p* = 0.023), with every 0.1 unit increase of FPG (OR = 1.22, *p* < 0.001), with every 0.1 unit increase of HbA1c (OR = 1.09, *p* = 0.001), and with every 1 unit increase of GA (OR = 1.22, *p* = 0.021) (Table 2).

**Table 2:** Regression analysis of factors associated with GDM

	Univariable		Multivariable	
	OR (95% CI)	*p* value	OR (95% CI)	*p* value
Age	1.11 (1.06, 1.15)	<0.001*	1.09 (1.04, 1.14)	<0.001*
BMI	1.08 (1.02, 1.13)	0.004*		
ALB	1.08 (1.02, 1.14)	0.007*	1.08 (1.01, 1.15)	0.023*
FPG	1.25 (1.18, 1.31)	<0.001*	1.22 (1.16, 1.29)	<0.001*
GA	1.27 (1.10, 1.47)	0.001*	1.22 (1.03, 1.44)	0.021*
HbA1c	1.13 (1.08, 1.19)	<0.001*	1.09 (1.03, 1.15)	0.001*
ALT	1.00 (0.99, 1.01)	0.907		
AST	1.00 (0.99, 1.01)	0.883		
TB	0.97 (0.92, 1.03)	0.295		
TBA	0.94 (0.87, 1.03)	0.196		
Urea	1.04 (0.95, 1.13)	0.392		
Cr	1.00 (0.97, 1.03)	0.814		
UA	1.00 (1.00, 1.00)	0.234		
TC	1.10 (0.92, 1.32)	0.282		
TG	1.41 (1.13, 1.76)	0.003*		
HDL-C	1.09 (0.70, 1.69)	0.699		
LDL-C	1.04 (0.83, 1.31)	0.736		
Hb	1.01 (0.99, 1.03)	0.375		
TP	1.04 (1.00, 1.08)	0.060		
AG	1.03 (0.91, 1.17)	0.674		
RBP4	1.04 (1.01, 1.06)	0.004*		

*AG* albumin/globulin ratio, *Alb* albumin, *Alt* alanine transaminase, *AST* aspartate aminotransferase, *BMI* body mass index, *CI* confidence interval, *Cr* creatinine, *FPG* fasting plasma glucose, *GA* glycated albumin, *GDM* gestational diabetes mellitus, *Hb* hemoglobin, *HbA1c* glycated hemoglobin, *HDL-C* high-density lipoprotein cholesterol, *LDL-C* low-density lipoprotein cholesterol, *OR* odds ratio, *RBP4* retinol conjugated protein 4, *TB* total bilirubin, *TBA* total bile acid, *TC* total cholesterol, *TG* triglycerides, *TP* total protein, *UA* uric acid, *BMI* body mass index, *GDM* gestational diabetes mellitus

*p < 0.05, indicates a significant association with GDM

### Influence of gestational weeks

**Fig. 2:**
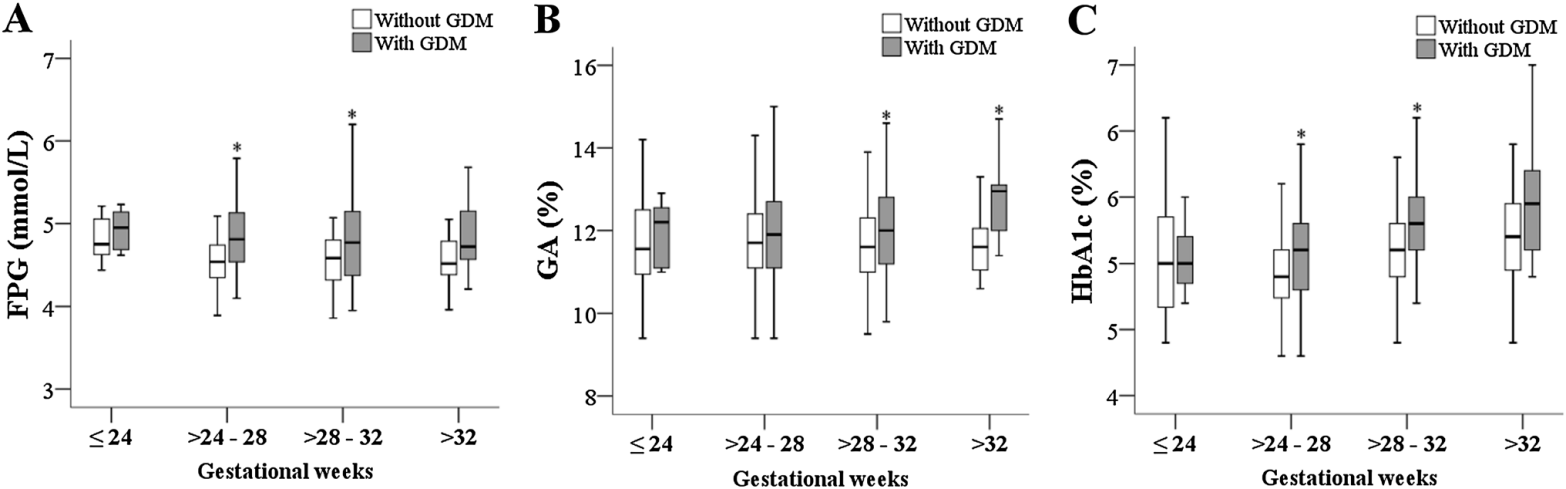
Differences in fasting plasma glucose (FPG) (**a**), glycated albumin (GA) (**b**), and glycated hemoglobin (HbA1c) (**c**) between pregnancies with and without gestational diabetes mellitus (GDM). Data are presented by median (*dashes* in the boxes), inter-quartile range (*boxes*), and full range (*whiskers*). *Indicates a significant difference between patients with and without GDM

**Fig. 3:**
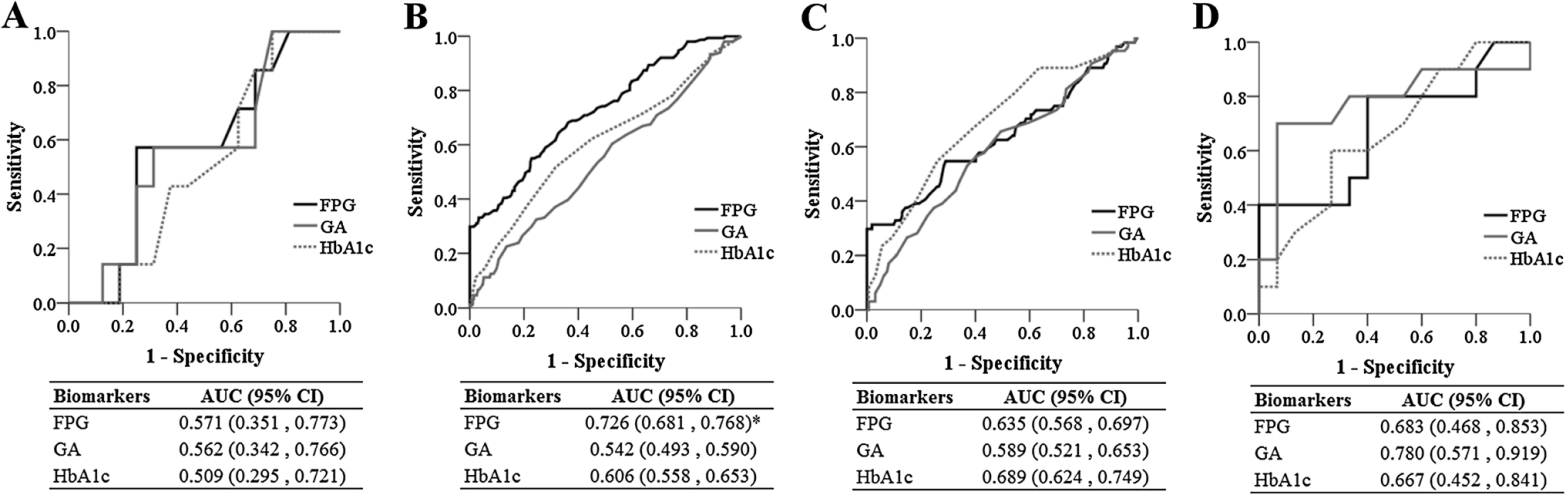
Diagnostic values of fasting plasma glucose (FPG), glycated albumin (GA), and glycated hemoglobin (HbA1c) by gestational weeks. **a** Less than 24 weeks’ gestation. **b** 24–28 weeks’ gestation. **c** 28–32 weeks’ gestation. **d** More than 32 weeks’ gestation. *The area under the receiver-operating characteristic curve (AUC) of FPG was significantly greater than those of GA and HbA1c. *CI* confidence interval

When the data were further stratified by gestational weeks, there were no significant differences in FPG, GA, and HbA1c between patients with and without GDM who were ≤24 weeks’ gestation. In patients 24–28 weeks’ gestation, those with GDM had significantly higher FPG and HbA1c levels; in patients 28–32 weeks’ gestation, those with GDM had significantly higher GA, FPG, and HbA1c levels; in patients >32 weeks’ gestation, those with GDM had a significantly higher GA level (Fig. 2). Diagnostic values of FPG, GA, and HbA1c stratified by gestational weeks are shown in Fig. 3. For pregnancies ≤24 weeks’ gestation (*n* = 23, Fig. 3a), the AUCs of FPG, GA, and HbA1c were not significantly higher than 0.5 (the 95% CIs contain 0.5), and were not different from each other. For pregnancies 24 to 28 weeks’ gestation (*n* = 424, Fig. 3b), the AUCs of FPG, GA, and HbA1c were significantly higher than 0.5, and the AUC of FPG was significantly higher than that of GA (0.726 vs. 0.542, *p* < 0.001) and HbA1c (0.726 vs. 0.606, *p* = 0.001). For pregnancies 28 to 32 weeks’ gestation (*n* = 226, Fig. 3c), the AUCs of FPG, GA, and HbA1c were significantly higher than 0.5. Although HbA1c had the highest AUC for detecting GDM, the differences in the AUCs of HbA1c, GA, and FPG did not obtain statistically significant. For pregnancies >32 weeks’ gestation (*n* = 25, Fig. 3d), only the AUC of GA obtained statistically significance (significantly higher than 0.5). The differences in the AUCs of HbA1c, GA, and FPG, however, were not statistically significant.

## Discussion

The results of this study showed that FPG has a higher diagnostic value than GA and HbA1c for the detection of GDM at 24–28 weeks’ gestation, and at gestational ages less than or greater than this range, the diagnostic value of FPG, GA, and HbA1c is similar. The final multivariable model showed that older age and higher levels of Alb, FPG, GA, and HbA1c were significantly associated with GDM. In addition, the results also show the baseline characteristics of age-matched control healthy women. This study can be further applied to the group of pregnant women with GDM or without GDM.

GA and HbA1c are the end-products of non-enzymatic glycosylation of the carboxyl groups of Alb and Hb, respectively [18, 19]. GA is believe to reflect more recent fluctuations of blood glucose than HbA1c, as the half-life of albumin is much shorter than that of hemoglobin [18, 19]. The diagnostic value of GA in non-pregnant individuals with DM has been confirmed by several studies [18–20], and correlations between GA and HbA1c have been determined [21]. Few studies, however, have been conducted to investigate the value of GA in patients with GDM, and the GA reference range in normal pregnant women has not been determined in all populations.

Pan et al. [22] studied women with GDM at 24–32 weeks’ gestation and reported that compared with HbA1c GA was more closely correlated with fasting and postprandial glucose levels regardless of insulin secretion and blood pressure. Hiramatsu et al. [24] determined reference intervals for GA and HbA1c in 574 pregnant Japanese women. The authors reported that HbA1c levels were significantly decreased in the second trimester of pregnancy and increased in the third trimester, GA levels significantly decreased towards the third trimester, and plasma glucose levels decreased in the first trimester and subsequently remained constant. The reference intervals of GA and HbA1c were 11.5–15.7 and 4.5–5.7%, respectively. GA levels were lower (*p* < 0.01) and HbA1c levels were higher (*p* < 0.05) in pregnant women with proteinuria, and in obese women, GA levels were lower (*p* < 0.01) than those of the control subjects (18.5 ≤ BMI < 25 kg/m^2^), and HbA1c levels were higher (*p* < 0.01). The results of the current study do not support these prior studies suggesting that GA may be a good index of GDM. One of the reasons may be a lack of control of variables that directly affect GA values during pregnancy, especially body weight. Stratification of patients into lean, normal, and obese groups based on body weight may demonstrate superiority of GA over HbA1c for the diagnosis of GDM. It is possible that GA may be less affected by factors, such as insulin resistance, and physiological fluctuations diastolic blood pressure during pregnancy, than HbA1c.

Even minor elevations of glucose in pregnancy are associated with worse fetal and maternal outcomes. Seabra et al. [25] performed a cross-sectional study of 829 healthy pregnant women and found that second and third trimester FPG levels below the cut-off values for a diagnosis of GDM were associated with an increased risk of pregnancy complications. In the current study, FPG provided a better diagnostic value than GA or HbA1c only at 24–28 weeks’ gestation. This result is consistent with a study by Trujillo et al. [26] who performed a multicenter cohort study of 4,926 pregnant women 20 years or older who received a single 2 h 75 g OGTT at 24–28 weeks gestation. A FPG cut-off value of 80 mg/dL indicated that only 38.7% of all women needed to undergo a complete OGTT, while detecting 96.9% of all GDM cases. When a cutoff of 85 mg/dL was used, the corresponding percentages were 18.7 and 92.5%, respectively. The authors concluded that using a FPG cutoff to diagnose GDM and to determine the need for and post-load OGTT measurements is a valid strategy to diagnose GDM. Trujillo et al. did not examine FPG levels at other time points in pregnancy, but based on the results of their study and ours, it appears that measurement of FPG is only useful when determined at 24–28 weeks’ gestation.

However, according to the current treatment guidelines for GDM, after excluding pre-pregnancy diabetes, GDM is diagnosed at 24 weeks or later. In the Trujillo study though, the OGTT was performed at 20 to 28 weeks’ gestation, and the data were collected from 1991 to 1995 which was before the current guidelines were published. In contrast, the current study was based on the most recent treatment guidelines.

## Strengths and limitations

There are a number of strengths as well as limitations of the current study. The sample size was relatively large, and the design effectively evaluated the value of GA, glycated hemoglobin, and FPG in the diagnosis of GDM. Moreover, we presented a normal reference of GA pregnant women without GDM corresponding to pregnancy trimester. With respect to limitations, the study was performed at a single institution and limited to a single ethnicity, and thus the results may not be generalizable to other populations. A non-pregnant control group was not included to determine the influences of pregnancy alone on GA; i.e., the design failed to exclude the possibility that pregnancy itself may cause an increase in GA. The design also did not allow comparison of GA, FPG, and glycated hemoglobin values in pregnant women without GDM in the first, second, and third trimesters of pregnancy. We did not evaluate the changed in GA from diagnosis of GDM to the period of glucose control to delivery. If this was done, we could evaluate the role of GA in blood glucose control in pregnant women with GDM. Metabolic factors, such as pre-pregnancy BMI and weight gain, during pregnancy were not examined as pre-pregnancy data were not available [27]. Finally, pregnancy outcomes were not available in several women, and GA was not measured in the third trimester of pregnancy. Thus, we could not evaluate the value of GA in the prediction of poor pregnancy outcomes women with and without GDM.

In conclusion, the results of this study do not support the use of GA as a screening tool for GDM. Further studies are necessary to determine the value of testing GA in pregnant women, and what factors may affect the results of testing.

## Electronic supplementary material

Below is the link to the electronic supplementary material.

Supplementary material 1 (DOCX 13 KB)

## References

[1] Mitanchez D (2010). Foetal and neonatal complications in gestational diabetes: perinatal mortality, congenital malformations, macrosomia, shoulder dystocia, birth injuries, neonatal complications. Diabetes Med.

[2] Rodríguez MM, López-Tinoco C, Murri M, Fernández-Deudero A, García-Palacios MV, García-Valero MA, Tinahones-Madueño FJ, Aguilar-Diosdado M, Roca (2014). Postpartum development of endothelial dysfunction and oxidative stress markers in women with previous gestational diabetes mellitus. J Endocrinol Invest.

[3] Bellamy L, Casas JP, Hingorani AD, Williams D (2009). Type 2 diabetes mellitus after gestational diabetes: A systematic review and meta-analysis. Lancet.

[4] Damm P (2009). Future risk of diabetes in mother and child after gestational diabetes mellitus. Int J GynecolObstet.

[5] Smyth S, Heron A (2005). Diabetes and obesity: The twin epidemics. Nat Med.

[6] Ferrara A (2007). Increasing prevalence of gestational diabetes mellitus: A public health perspective. Diabetes Care.

[7] Metzger BE, Gabbe SG, Persson B, Buchanan TA, Catalano PA, Damm P, Dyer AR, Leiva Ad, Hod M, Kitzmiler JL, Lowe LP, McIntyre HD, Oats JJ, Omori Y, Schmidt MI, International Association of Diabetes and Pregnancy Study Groups Consensus Panel (2010). International association of diabetes and pregnancy study groups recommendations on the diagnosis and classification of hyperglycemia in pregnancy. Diabetes Care.

[8] Agarwal MM, Shah SM, Al Kaabi J, Saquib S, Othman Y (2015). Gestational diabetes mellitus: Confusion among medical doctors caused by multiple international criteria. J ObstetGynaecol Res.

[9] Scifres CM, Abebe KZ, Jones KA, Comer DM, Costacou T, Simhan HN, Day NL, Davis EM, Freiberg MS (2015). Gestational diabetes diagnostic methods (GD2M) pilot randomized trial. Matern Child Health J.

[10] McIntyre HD, Dyer AR, Metzger BE (2015). Odds, risks and appropriate diagnosis of gestational diabetes. Med J Aust.

[11] World Health Organization (2013). Diagnostic criteria and classification of hyperglycaemia first detected in pregnancy.

[12] Harrison CL, Lombard CB, East C, Boyle J, Teede HJ (2015). Risk stratification in early pregnancy for women at increased risk of gestational diabetes. Diabetes Res ClinPract.

[13] American Diabetes Association (2011). Standards of medical care in diabetes–2011. Diabetes Care.

[14] Verhaeghe J, Van Herck E, Benhalima K, Mathieu C (2012). Glycated hemoglobin in pregnancies at increased risk for gestational diabetes mellitus. Eur J ObstetGynecolReprodBiol.

[15] Nagalla SR, Snyder CK, Michaels JE, Laughlin MJ, Roberts CT, Balaji M, Balaji V, Seshiah V, Rao PV (2015). Maternal serum biomarkers for risk assessment in gestational diabetes. A potential universal screening test to predict GDM status. Indian J EndocrinolMetab.

[16] Hashimoto K, Osugi T, Noguchi S, Morimoto Y, Wasada K, Imai S, Waguri M, Toyoda R, Fujita T, Kasayama S, Koga M (2010). A1c but not serum glycated albumin is elevated because of iron deficiency in late pregnancy in diabetic women. Diabetes Care.

[17] Mosca A, Paleari R, Dalfrà MG, Di Cianni G, Cuccuru I, Pellegrini G, Malloggi L, Bonomo M, Granata S, Ceriotti F, Castiglioni MT, Songini M, Tocco G, Masin M, Plebani M, Lapolla A (2006). Reference intervals for hemoglobin A1c in pregnant women: data from an Italian multicenter study. ClinChem.

[18] Lee JE (2015). Alternative biomarkers for assessing glycemic control in diabetes: fructosamine, glycated albumin, and 1,5-anhydroglucitol. Ann PediatrEndocrinolMetab.

[19] Ikezaki H, Furusyo N, Ihara T, Hayashi T, Ura K, Hiramine S, Mitsumoto F, Takayama K, Murata M, Kohzuma T, Ai M, Schaefer EJ, Hayashi J (2015). Glycated albumin as a diagnostic tool for diabetes in a general Japanese population. Metabolism.

[20] Ma XJ, Pan JM, Bao YQ, Zhou J, Tang JL, Li Q, Xiang KS, Jia WP (2010). Combined assessment of glycated albumin and fasting plasma glucose improves the detection of diabetes in Chinese subjects. ClinExpPharmacolPhysiol.

[21] Inoue K, Tsujimoto T, Yamamoto-Honda R, Goto A, Kishimoto M, Noto H, Kajio H, Doi S, Miyazaki S, Terauchi Y, Noda M (2014). A newer conversion equation for the correlation between HbA1c and glycated albumin. Endocr J.

[22] Pan J, Zhang F, Zhang L, Bao Y, Tao M, Jia W (2013). Influence of insulin sensitivity and secretion on glycated albumin and hemoglobin A1c in pregnant women with gestational diabetes mellitus. Int J GynaecolObstet.

[23] Hashimoto K, Koga M (2015). Indicators of glycemic control in patients with gestational diabetes mellitus and pregnant women with diabetes mellitus. World. J Diabetes.

[24] Hiramatsu Y, Shimizu I, Omori Y, Nakabayashi M, JGA (Japan Glycated Albumin) Study Group (2012). Determination of reference intervals of glycated albumin and hemoglobin A1c in healthy pregnant Japanese women and analysis of their time courses and influencing factors during pregnancy. Endocr J.

[25] Seabra G, Saunders C, de CarvalhoPadilha P, Zajdenverg L, da Silva LB, de Souza Santos MM (2015). Association between maternal glucose levels during pregnancy and gestational diabetes mellitus: an analytical cross-sectional study. DiabetolMetabSyndr.

[26] Trujillo J, Vigo A, Reichelt A, Duncan BB, Schmidt MI (2014). Fasting plasma glucose to avoid a full OGTT in the diagnosis of gestational diabetes. Diabetes Res ClinPract.

[27] Huang Y, Hu Y, Ma YU, Ye G (2015). Glycated albumin is an optimal biomarker for gestational diabetes mellitus. ExpTher Med.

