# Supplementary material for: The diagnostic value of glycated albumin in gestational diabetes mellitus
Source: J Endocrinol Invest. 2017 Jun 6;41(1):121–8. doi: 10.1007/s40618-016-0605-7 (PMC5754373; doi:10.1007/s40618-016-0605-7)
Supplement: Supplementary file 1 — Supplementary material 1 (DOCX 13 KB) [file 40618_2016_605_MOESM1_ESM.docx]

Supplemental Table 1. Weight gain of women with and without GDM at different points in pregnancy

|  | Without GDM | With GDM | *p*-value |
| --- | --- | --- | --- |
| Weight (kg) |  |  |  |
| Pre-pregnancy | 53.99±8.41 | 56.21±8.97 | 0.001 |
| Week 13-24 | 58.89±9.25 | 61.14±9.09 | 0.003 |
| Week 24-28 | 62.27±8.98 | 64.44±8.86 | 0.003 |
| Week 28-32 | 64.45±8.94 | 66.59±9.04 | 0.006 |
| Week 32-36 | 66.18±8.86 | 68.58±8.92 | 0.004 |
| Week 36-38 | 67.75±9.04 | 70.37±9.2 | 0.002 |
| Week 38-labor | 70.45±31.56 | 72.02±9.21 | 0.572 |
| BMI |  |  |  |
| Pre-pregnancy | 20.85±3.06 | 21.8±3.39 | <0.001 |
| Week 13-24 | 22.67±3.32 | 23.53±3.19 | 0.002 |
| Week 24-28 | 24.01±3.22 | 24.87±3.22 | 0.001 |
| Week 28-32 | 24.81±3.23 | 25.71±3.2 | 0.001 |
| Week 32-36 | 25.46±3.18 | 26.47±3.2 | 0.001 |
| Week 36-38 | 26.05±3.27 | 27.19±3.3 | <0.001 |
| Week 38-labor | 27±11.96 | 27.69±3.16 | 0.508 |
| Weight gain from pre-pregnancy | |  |  |
| Week 13-24 | 4.94±4.71 | 5.21±3.75 | 0.47 |
| Week 24-28 | 8.39±3.88 | 8.61±3.82 | 0.481 |
| Week 28-32 | 10.29±3.49 | 10.35±3.61 | 0.847 |
| Week 32-36 | 11.91±3.55 | 12.16±3.91 | 0.469 |
| Week 36-38 | 13.33±3.63 | 13.76±3.86 | 0.214 |
| Week 38-labor | 15.84±30.77 | 15.01±3.76 | 0.755 |

BMI, body mass index; GDM, gestational diabetes mellitus.
